# Serum beta-2 microglobulin as a predictor of nephritis, disease activity, and damage score in systemic lupus erythematosus: a cross-sectional study

**DOI:** 10.1007/s00296-022-05221-1

**Published:** 2022-10-07

**Authors:** Dalia Mohamed Gamal, Fatma Mohammed Badr, Sara Ibrahim Abd el Fattah Taha, Nouran M. Moustafa, Mohammed Abd El Monem Teama

**Affiliations:** 1 Internal Medicine, Rheumatology and Immunology, Faculty of Medicine, 2 Staff Member Buildings of Ain Shams University, Ramsis Street, Abbassya Square, El Al Waili/El-Abaseya, Post No: 11517, Cairo, Egypt; 2 Clinical Pathology Department, Faculty of Medicine, Ain Shams University, Ramsis Street, Abbassya Square, Cairo, Egypt; 3 Medical Microbiology and Immunology Department, Faculty of Medicine, Ain Shams University, Ramsis Street, Abbassya Square, Cairo, Egypt

**Keywords:** Serum b2microglobulin, Lupus nephritis, Disease activity, Biomarker, Prognosis

## Abstract

A strong correlation between lupus nephritis (LN), disease activity, and serum beta 2-microglobulin (b2MG) was observed. The current study examines the correlation between serum b2MG and renal involvement, damage score, and disease activity in systemic lupus erythematosus (SLE) patients. One hundred SLE patients from Ain Shams University Hospital were enrolled and categorized into two groups. Group I had 40 patients with negative b2MG, while Group II had 60 patients with positive b2MG levels. Medical history, clinical examination, and assessing disease activity based on SLE disease activity index (SLEDAI-2 K), and damage score were recorded for all patients. Laboratory examinations, such as serum b2MG, complete blood count, blood urea nitrogen (BUN), serum creatinine, glomerular filtration rate (GFR), urine analysis, 24 h urinary protein excretion, Antinuclear antibodies (ANA), anti-dsDNA antibody, and serum complement (C3, C4). BUN, 24 h urinary protein, serum creatinine, active urinary sediment, SLEDAI score, and damage score were all elevated in group II compared to group I (*p* < 0.001). There is a positive correlation between serum b2MG and 24 h urinary protein, BUN, serum creatinine, disease activity, and damage score (*p* < 0.001), while it was negatively correlated with GFR, C3, and C4 (*p* < 0.001). Serum b2MG has proven to be a predictor of LN in SLE patients (Sensitivity 92.45%, Specificity 74.47%), also being a predictor of the activity of the disease as well as damage index (Sensitivity 96.67%, Specificity 85%) (Sensitivity 92.45%, Specificity 74.47%), respectively. Serum b2MG level can be used as a valuable predictor for LN, clinical disease activity, and damage score.

## Introduction

Systemic lupus erythematosus (SLE) is a chronic autoimmune disease, which primarily affects young women. It is characterized by the formation of autoantibodies against cytoplasmic and nuclear antigens. Though its etiology is unknown, several genetic, epigenetic, and environmental risk factors have been identified [1].

Around 60–80% of SLE cases present variable degrees of renal involvement in the form of Lupus nephritis (LN). Common symptoms include renal insufficiency, proteinuria, hematuria, and irreversible renal failure in about 10–20% of the cases [2]. LN is responsible for elevated mortality and morbidity in lupus patients, especially when combined with treatment side effects of renal diseases. Hence, it is critical to evaluate the activity for early detection of renal involvement in lupus cases [3]. Individualized treatment should be provided to SLE patients based on the degree of renal involvement. Constant fluctuations in autoantibodies and other complement levels are induced by the alternating incidence of active and stable disease in patients with SLE [4]. Therefore, the identification of these indices for assessing LN and disease activity has significant clinical consequences [5].

Beta-2 microglobulin (b2MG), an 11 kDa molecular weight protein, is linked to the light chain of major histocompatibility complex class I (MHC-I) antigens. It is mainly secreted by the immune-associated cells, such as activated T-cells, macrophages, and B-lymphocytes [6]. B2MG plays a vital role in immune responses in various autoimmune diseases. Leffers et al. reported that a higher b2MG was observed in SLE patients than in the general population [7]. Several studies suggest that serum b2MG might be an effective indicator to assess SLE activity [8–10]. B2MG levels in patients with LN have been significantly correlated with renal disease activity [11]. Thus, serum b2MG can be used as a monitoring parameter for diagnosing renal damage and early prognosis of further renal diseases [12].

Our study examines the association of serum b2MG with renal involvement, disease activity, and damage score in SLE patients and evaluates its validity as a predictor of LN.

## Materials and methods

### Patients

This cross-sectional study recruited 100 SLE patients, age > 18 years, based on the Systemic Lupus International Collaborating Clinics classification criteria [13], who attended Ain Shams University Hospitals from July 2021 to February 2022. The patients were categorized into two groups based on b2MG levels; group I (40 patients with negative serum b2MG) and group II (60 patients with positive serum b2MG).

Patients with a history of a bacterial or viral infection 3 months before enrollment; patients with hypertension, other cardiovascular diseases, malignant tumors, rheumatoid arthritis, central system diseases, diabetes, liver diseases; patients under immunosuppressive medicines or any drugs that impact serum creatinine or b2MG levels; patients suffering from renal damage due to other factors were excluded from the study.

### Ethical considerations

This study was performed according to the criteria described in the World Medical Association's Helsinki Declaration after due approval from the local ethics committee of Ain Shams University. The reference number of the study was FWA00001786 on 25 May 2021. Written informed consents obtained from all subjects.

### Methods

The sex, age, duration of the disease, and clinical and rheumatological history of the patients were recorded. Laboratory investigations were conducted, including C-reactive protein (CRP) by Cobas C6000 autoanalyzer (Roche Diagnostics GmbH, Mannheim, Germany), kidney function tests (BUN, serum creatinine) by AU680 Beckman Coulter autoanalyzer (Beckman Coulter, Inc., Brea, CA), complete blood count (CBC) by Sysmex XT-1800i autoanalyzer (Sysmex, Japan), erythrocyte sedimentation rate (ESR) by Westergren method, and routine urine analysis besides assessing active urinary sediments (white blood cells, red blood cells, and casts or proteins). In addition, 24-h urine protein excretion and eGFR were estimated using the original Modification of Diet in Renal Disease (MDRD) equation [14]:

$${\text{eGFR}}\left( {{\text{ml}}/\min /1.73\;{\text{m}}^{2} } \right) = 186 \times \left( {{\text{Creat}}/88.4} \right)^{ - 1.154} \times \left( {{\text{Age}}} \right)^{ - 0.203} \times \left( {0.742\;{\text{if}}\;{\text{female}}} \right) \times \left( {1.210\;{\text{if}}\;{\text{black}}} \right)$$

Renal biopsy was performed by the assessment of Activity ibex (AI) and chronicity index (CI) of the LN patients. Histopathological classification of LN was conducted based on the revised classification of the International Society of Nephrology and the Renal Pathology Society [15]. STA Compact Max® coagulation analyzer (Stago, USA) studied Lupus anticoagulant. (DiaSorin, USA) analyzed ANA and anti-dsDNA antibodies using an indirect immunofluorescence technique with commercial slides. Anti-cardiolipin (IgG, IgM) levels were studied by ELISA (Invitrogen, Fisher Scientific, USA). Serum complements (C3, C4) were evaluated by Cobas C6000 autoanalyzer (Roche Diagnostics GmbH, Mannheim, Germany).

A sterile venipuncture was used to collect the peripheral venous blood sample from each participant. It was then placed in a simple vacutainer tube with a clot activator for serum separation. Clotting of blood was allowed for 30 min prior to centrifugation at 3000*g* for 15 min. Separated sera was kept at – 80 ℃ until further analysis. A commercially available human ELISA kit was used for b2MG determination (ORGENTEC Diagnostika GmbH, Mainz, Germany). The detection range of the ELISA assay was 0–3 µg/ml, with a sensitivity of 0.1 µg/ml. Patients who had serum b2MG ≥ 3 µg/ml were reported positive for the marker [16].

### Assessment of disease activity

Assessment of SLE disease activity was done based on SLEDAI-2 K [17]. SLEDAI scores were categorized as follows:

- SLEDAI = 20 indicates very high activity,
- SLEDAI = 11–19 indicates high activity,
- SLEDAI = 6–10 indicates moderate activity,
- SLEDAI = 1–5 indicates mild activity,
- SLEDAI = 0 indicates no activity.

### Assessment of SLE damage

The SLICC/ACR Damage Index (SDI) was incorporated for measuring irreversible cumulative damage induced by the disease activity in SLE. SDI includes parameters that signify persistent, irreversible impairment in the lupus patients. Apart from stroke and myocardial infarction, most indications have to last at least 6 months. Damage is considered for the following twelve systems: malignancies (0–2), gonadal (0–1), endocrine (diabetes) (0–1), skin (0–3), musculoskeletal (0–7), gastrointestinal (0–6), peripheral vascular (0–5), cardiovascular (0–6), pulmonary (0–5), renal (0–3), neuropsychiatric (0–6), and ocular (range 0–2). Damage may be either steady or incremental with time (up to a theoretical maximum of 47 points) [18].

### Statistical analysis

SPSS software, v. 25 was used to perform all statistical analyses of the collected data. Parameters were analyzed according to the data type. Normality tests were conducted to check the data distribution (Kolmogorov–Smirnov, and Shapiro–Wilk tests). Qualitative results were represented as frequencies and percentages and quantitative data were represented as mean ± standard deviation, range, and median values. While unpaired Student’s *t* test was used to compare the quantitative data of the two groups, chi-square test was used to examine the relationship between their qualitative variables. Mann–Whitney Test (*U* test) was used for nonparametric variables. Fisher's exact test was incorporated to determine the association between the qualitative variables in case the expected count was lower than five in more than 20% of cells. The correlations between b2MG level and other parameters were examined using the Spearman rank correlation coefficient.

A Receiver Operating Characteristic (ROC) curve analysis was conducted to examine the diagnostic value of b2MG. The area under the ROC curve (AUC) for the diagnostic/predictive value was interpreted as follows: excellent: 0.9–1.0; good: 0.8–0.89; fair: 0.7–0.79; poor: 0.6–0.69; and fail: < 0.6). Specificity was expressed as a percentage. The level of significance was determined from the *P* values as follows:

- *P* > 0.050: non-significant (NS).
- *P* < 0.050: significant (S).
- *P* ≤ 0.001: highly significant (HS).

## Results

The demographic data of the groups were compared. The difference in age and duration of disease was insignificant between the groups. However, the composition of sex varied between the groups significantly, as shown in Table 1. Group II patients exhibited more significant clinical characteristics, such as oral ulcer, serositis (either pleural or pericardial effusion), nephritis, neurological, cardiac, pulmonary, and hematological manifestations than group I patients. Furthermore, group II patients also had significantly heightened pathological conditions than group I, including anemia, ESR, CRP, and different parameters of LN, including (24 h urinary protein, active urinary sediment including casts, hematuria, pyuria, albuminuria, BUN, and serum creatinine). Consumed C3 and C4 values were also higher, while eGFR was highly significant lower in-group II patients. Several renal biopsy classes were found to be elevated in-group II and the patients also had significantly higher SLEDAI 2 K and damage scores than group I. Neurological, renal, and cardiac damage seemed to be more significantly prevalent in-group II than in-group I, as depicted in Table 2. Evidently, serum b2MG was positively correlated with 24-h urinary protein, BUN, serum creatinine, ESR, CRP, SLEDAI 2 K, and damage score. However, it had a negative correlation to hemoglobin (HGB), GFR, and consumed C3 and C4 levels. No substantial relationship was observed between serum b2MG and many other parameters in the study, such as WBC, neutrophils, lymphocyte, and platelet counts (Table 3).

**Table 1:** Comparison between two groups as regards demographic data, clinical manifestations, laboratory data, and disease activity

Demographic data, clinical manifestations & laboratory data and disease activity	B2 microglobulin		Value	*P* value
	Group I	Group II		
	Mean ± SD Median (IQR) *N* (%)	Mean ± SD Median (IQR) *N* (%)		
Age (years)	28.2 ± 7.44	30.2 ± 9.24	*t*** = **− 1.143	0.256
Sex				
Male	3 (14.29%)	18 (85.71%)	*X*^2^** = **7.324	0.007*
Female	37 (46.84%)	42 (53.16%)		
Disease duration (years)	3 (2–5)	4 (2–6)	*z*** = **− 0.722	0.471
Malar rash				
No	3 (60%)	2 (40%)	Fisher's Exact test	0.386
Yes	37 (38.95%)	58 (61.05%)		
Oral ulcer				
No	9 (69.23%)	4 (30.77%)	*X*^2^** = **5.32	0.021*
Yes	31 (35.63%)	56 (64.37%)		
Musculoskeletal manifestations				
No	1 (50%)	1 (50%)	Fisher's Exact test	0.178
Arthralgia	33 (44.59%)	41 (55.41%)		
Arthritis	6 (25%)	18 (75%)		
Photosensitivity				
No	20 (39.22%)	31 (60.78%)	*X*^2^** = **0.027	0.87
Yes	20 (40.82%)	29 (59.18%)		
Alopecia				
No	19 (38.78%)	30 (61.22%)	*X*^2^** = **0.06	0.806
Yes	21 (41.18%)	30 (58.82%)		
Constitutional symptoms				
No	9 (45%)	11 (55%)	*X*^2^** = **0.26	0.61
Yes	31 (38.75%)	49 (61.25%)		
Serositis				
No	31 (50.82%)	30 (49.18%)	*X*^2^** = **7.629	0.006*
Yes	9 (23.08%)	30 (76.92%)		
Neurological manifestations				
No	38 (48.72%)	40 (51.28%)	*X*^2^** = **11.228	0.001*
Yes	2 (9.09%)	20 (90.91%)		
Nephritis				
No	35 (74.5%)	12 (25.5%)	*X*^2^** = **43.898	< 0.001*
Yes	5 (9.4%)	48 (90.6%)		
Recurrent Thrombosis				
No	35 (39.77%)	53 (60.23%)	Fisher's Exact test	1.00
Yes	5 (41.67%)	7 (58.33%)		
Bleeding tendency				
No	35 (37.23%)	59 (62.77%)	Fisher's Exact test	0.036*
Yes	5 (83.33%)	1 (16.67%)		
Cardiac manifestation				
No	37 (46.25%)	43 (53.75%)	*X*^2^** = **6.51	0.011*
Yes	3 (15%)	17 (85%)		
Pulmonary manifestations				
No	33 (49.25%)	34 (50.75%)	*X*^2^** = **7.244	0.007*
Yes	7 (21.21%)	26 (78.79%)		
Haematological manifestations				
No	25 (50%)	25 (50%)	*X*^2^** = **4.167	0.041*
Yes	15 (30%)	35 (70%)		
WBC (109/L)	5 (4–6.4)	4.8 (3.2–6.1)	*z*** = **− 0.790	0.430
Neutrophils (109/L)	4118.75 ± 1721.2	4163.33 ± 1849.96	*t*** = **− 0.121	0.904
Lymphocytes (109/L)	1250 (1100–2000)	1200 (900–2000)	*z*** = **− 0.813	0.416
HGB (gram /dl)	11.21 ± 1.34	9.9 ± 1.48	*t*** = **4.491	< 0.001*
Platelet (109/L)	226.13 ± 82.78	234.7 ± 79.26	*t*** = **− 0.521	0.604
ESR (mm/hr)	30 (20–35)	58 (40–60)	*z*** = **− 5.117	< 0.001*
CRP (mg/L)	6 (4–6)	12 (6–48)	*z*** = **− 5.389	< 0.001*
Urine analysis				
Albuminuria by dipstick				
No	35 (72.92%)	13 (27.08%)	*X*^2^** = **41.673	< 0.001*
Yes	5 (9.62%)	47 (90.38%)		
Urinary Cast				
No	40 (48.78%)	42 (51.22%)	*X*^2^** = **14.634	< 0.001*
Yes	0 (0%)	18 (100%)		
Heamturia (> 5/HPF)				
No	40 (45.45%)	48 (54.55%)	Fisher’s Exact test	0.001*
Yes	0 (0%)	12 (100%)		
Pyuria (> 5/HPF)				
No	39 (45.35%)	47 (54.65%)	*X*^2^** = **7.323	0.007*
Yes	1 (7.14%)	13 (92.86%)		
24 h urinary protein (mg/24 h)	150 (100–200)	900 (350–2000)	*z*** = **− 6.555	< 0.001*
Bun (mg/dl)	18 (18–20)	20 (18–22)	*z*** = **− 3.014	0.003*
Serum creatinine (mg/dl)	0.55 (0.5–0.7)	0.7 (0.5–1.8)	*z*** = **− 2.903	0.004*
G.F.R (ml/min)	89.1 ± 7.37	75.38 ± 23.43	*t*** = **4.232	< 0.001*
Consumed C3	121 (92.5–135)	70.5 (62–121)	*z*** = **− 4.036	< 0.001*
Consumed C4	22 (14–23)	8 (6–20)	*z*** = **− 4.874	< 0.001*
LAC Abs		`		
Negative	34 (37.36%)	57 (62.64%)	Fisher's Exact test	0.151
Positive	6 (66.67%)	3 (33.33%)		
A.C.L Abs				
Negative	37 (39.78%)	56 (60.22%)	Fisher's Exact test	1.00
Positive	3 (42.86%)	4 (57.14%)		
Renal biopsy				
No	38 (54.3%)	32 (45.7%)	*X*^2^** = **19.841	< 0.001*
Yes	2 (6.7%)	28 (93.3%)		
Renal biopsy Classes				
II	1 (50%)	1 (50%)	Fisher's Exact test	0.168
III	1 (11.11%)	8 (88.89%)		
IV	0 (0%)	6 (100%)		
V	0 (0%)	10 (100%)		
VI	0 (0%)	3 (100%)		
Activity index	4 (1–6)	7(6–8)	*z*** = **− 1.07	0.092
Chronicity index	2 (1–3)	2 (1–3)	*z*** = **− 0.04	0.965
Anti- ds DNA				
Negative	4 (50%)	4 (50%)	Fisher's Exact test	0.71
Positive	36 (39.13%)	56 (60.87%)		
ANA				
Negative	1 (100%)	0 (0%)	Fisher's Exact test	0.4
Positive	39 (39.39%)	60 (60.61%)		
SLEDAI 2 K SCORE	0 (0–1)	7 (6–10)	*z*** = **− 7.221	< 0.001*

*P* value > 0.05: non-significant (NS); *P *value < 0.05: significant (S); *P* value < 0.01: highly significant (HS)

*ESR* erythrocyte sedimentation rate, *CRP* C-reactive protein, *Bun* blood urea nitrogen, *GFR* glomerular filtration rate, *C3* complement 3, *C4* complement 4, *LAC* lupus anticoagulant, *ACL* Anticardiolipin, *ANA* antinuclear antibody, *SLEADI score* systemic lupus erythematosus disease activity

*Significant test

**Table 2:** Comparison between two groups as regards Damage index parameters and score

Damage index parameters and score	B2 Microglobulin		Value	*p* value
	Group I *N *= 40	Group II *N* = 60		
	*N* (%) Median (IQR)	*N* (%) Median (IQR)		
Ocular damage				
No	40 (40.4%)	59 (59.6%)	Fisher's Exact test	1.00
Yes	0 (0%)	1 (100%)		
Neurological damage				
0	38 (49.35%)	39 (50.65%)	Fisher's Exact test	< 0.001*
1	1 (5.26%)	18 (94.74%)		
2	1 (33.33%)	2 (66.67%)		
Pulmonary damage				
No	40 (41.24%)	57 (58.76%)	Fisher's Exact test	0.273
Yes	0 (0%)	3 (100%)		
Cardiac damage				
No	40 (45.98%)	47 (54.02%)	Fisher's Exact test	0.009*
Yes	0 (0%)	9 (100%)		
Renal damage				
0	40 (47.62%)	44 (52.38%)	Fisher's Exact test	0.001*
1	0 (0%)	13 (100%)		
2	0 (0%)	1 (100%)		
3	0 (0%)	2 (100%)		
Vascular damage				
No	38 (38.78%)	60 (61.22%)	Fisher's Exact test	0.158
Yes	2 (100%)	0 (0%)		
Endocrinal damage				
No	39 (41.49%)	55 (58.51%)	Fisher's Exact test	0.397
Yes	1 (16.67%)	5 (83.33%)		
Gastrointestinal damage				
No	40 (40%)	60 (60%)		
Yes	0 (0%)	0 (0%)		
Musculoskeletal damage				
No	40 (40%)	60 (60%)		
Yes	0 (0%)	0 (0%)		
Gonadal damage				
No	40 (40%)	60 (60%)		
Yes	0 (0%)	0 (0%)		
Skin damage				
No	40 (40%)	60 (60%)		
Yes	0 (0%)	0 (0%)		
Total score (damage score)	0 (0–0)	1 (0–1.5)	*z* **= **− 5.525	< 0.001*

*P* value > 0.05: non-significant (NS); *P* value < 0.05: significant (S); *P* value < 0.01: highly significant (HS)

*Significant test

**Table 3:** Correlation between serum B2 microglobulin and different laboratory findings, disease activity and damage score

B2 microglobulin level	Spearman's rho	*p* value
WBCs (109/L)	− 0.119	0.237
Neutrophils (109/L)	− 0.046	0.652
Lymphocytes (109/L)	− 0.150	0.136
HGB (gram/dl)	− 0.446	< 0.001*
Platelet (109/L)	− 0.101	0.317
ESR (mm/hr)	0.499	< 0.001*
CRP (mg/L)	0.479	< 0.001*
24 h urinary protein (mg/24 h)	0.733	< 0.001*
Bun (mg/dl)	0.351	< 0.001*
Serum creatinine (mg/dl)	0.373	< 0.001*
G.F.R (ml/min)	− 0.32	0.001*
C3 (mg/dl)	− 0.409	< 0.001*
C4 (mg/dl)	− 0.445	< 0.001*
SLEDAI 2 K SCORE	0.725	< 0.001*
Total score (damage score)	0.603	< 0.001*
Activity index	− 0.029	0.880
Chronicity index	0.174	0.359

*P* value > 0.05: non-significant (NS); *P* value < 0.05: significant (S); *P* value < 0.01: highly significant (HS)

*WBC* white blood cell**, ***ESR* erythrocyte sedimentation rate, *CRP* C-reactive protein, *Bun* blood urea nitrogen, *GFR* glomerular filtration rate, *C3* complement 3, *C4* complement 4, *SLEADI score* systemic lupus erythematosus disease activity

*Significant test

**Fig. 1:**
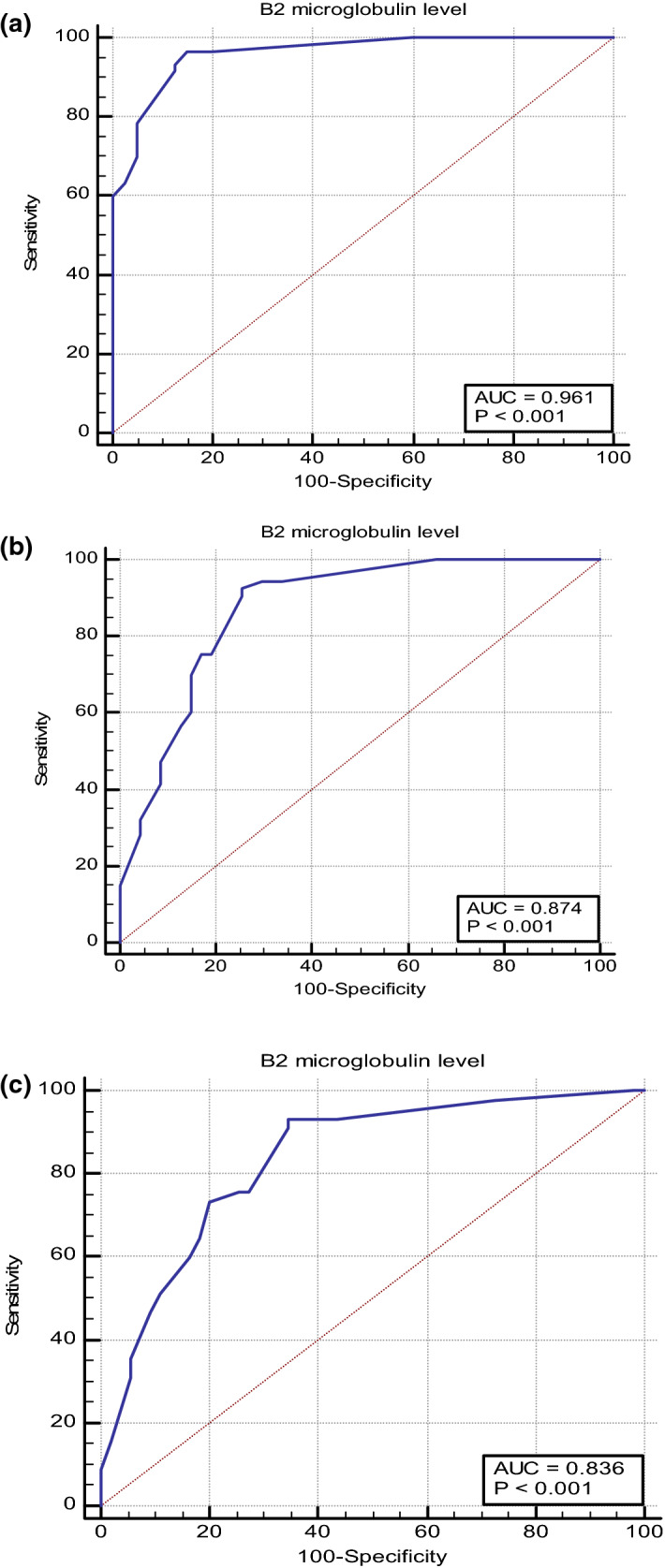
**a** Serum B2 microglobulin as a predictor of disease activity (SLEDAI). **b **Serum B2 microglobulin as a predictor of lupus nephritis. **c** Serum B2 microglobulin as a predictor of damage

A ROC curve was plotted, and its AUC was determined to evaluate the diagnostic value of serum b2MG in LN and identify its role as a marker of damage index and disease activity. Cutoff values and performance characteristics are presented in Table 4, Fig. 1a–c.

**Table 4:** Serum B2 microglobulin as a predictor of disease activity (SLEDAI), damage, and lupus nephritis

Parameter	AUC	95% CI	significant	Cutoff value	Sensitivity	Specificity	Positive predictive value	Negative predictive value
B2 microglobulin µg/ml/SLEDAI score)	0.961	0.902–0.99	< 0.001*	> 2.2	96.67	85	90.6	94.4
B2 microglobulin (µg/ml)/damage	0.836	0.749–0.903	< 0.001*	> 2.6	93.33	65.45	68.9	92.3
B2 microglobulin (µg/ml)/nephritis	0.874	0.793–0.932	< 0.001*	> 2.6	92.45	74.47	80.3	89.7

*P* value > 0.05: non-significant (NS); *P* value < 0.05: significant (S); *P* value < 0.01: highly significant (HS)

*AUC* area under curve, *SLEADI score* systemic lupus erythematosus disease activity

*Significant test

Serum b2MG value of > 2.6 µg/ml was predictive of LN with 74.47 specificity, 92.45 sensitivity, 80.3 positive predictive value (PPV), 89.7 negative predictive value (NPV), and 0.874 AUC. Meanwhile, serum b2MG, a value of > 2.2 µg/ml was predictive of disease activity with a specificity of 85, sensitivity of 96.67, PPV of 90.6, NPV of 94.4, and an AUC of 0.961. As a predictor of damage, serum b2MG performed as follows: the cutoff value was > 2.6 µg/ml with 65.45 specificity, 93.33 sensitivity, 68.9 PPV, 92.3 NPV, and an AUC of 0.836.

## Discussion

Systemic lupus erythematosus (SLE) is an illness of diffuse connective tissue concerning many organs, which manifests due to deficiency in immunological tolerance. Its primary pathogenic characteristics include autoantibody secretion, deposition, and aggregation of immune complexes [19]. Complications in SLE arise due to renal involvement, with as much as 85% of SLE patients developing LN over their lifetime. Currently, a noninvasive approach for assessing the degree of renal injury is required for early and easy detection of LN. Furthermore, for proper management, prediction of clinical disease activity is also necessary [20].

It is evident that b2MG contributes to SLE pathogenesis, suggesting that the protein is involved in the increased secretion of autoantibodies leading to hypergammaglobulinemia [21]. Several studies have reported increased b2MG in urine and serum of SLE patients, which is significantly correlated with LN and its clinical disease activity [10, 11]. The current study included 100 SLE patients to examine the role of serum b2MG in the prediction of LN development, clinical disease activity, and overall damage score.

Both study groups substantially differed in sex, with group II being substantially higher (*p* < 0.05). Nevertheless, there was no difference between both groups concerning age and disease duration (*p* > 0.05). Choe et al. demonstrated that age and duration of disease were not markedly correlated with serum b2MG (*p* > 0.05) [22]. Other parameters such as oral ulcer, serositis, neurological, cardiac, pulmonary, and nephritis were presented more in-group II than in-group I (*p* < 0.05). These results align with the study by Kim et al., who showed that SLE cases with serositis, nephritis, and oral ulcer had markedly elevated levels of b2MG compared to those without them. However, arthritis was the only exception [10]. Contrarily, Abd-Elbaky et al. reported increased serum b2MG in SLE cases with arthritis, cutaneous and/or mucosal manifestations, Lupus nephritis, and cardiac manifestations but not in cases experiencing hematological, neurological, or ocular manifestations [23].

Furthermore, the current study revealed a significant difference in 24 h urinary protein excretion, serum creatinine, BUN, eGFR, active urinary sediment, albuminuria, consumed C3, C4 ( which are parameters of lupus nephritis), ESR, and CRP between the two groups with significantly elevated levels in group II (*p* < 0.05). These results are in accordance with those of Choe et al*.,* who revealed a positive relationship between serum b2MG and protein/creatinine ratio, ESR, and BUN, but not with an anti-dsDNA titre, complements, or CRP [22]. Badr et al. also showed that b2MG is a sensitive and precise indicator of renal action. In addition, they detected a significant correlation between GFR and serum b2MG levels [24]. Skare et al*. *assessed the link between CRP, ESR, and serum b2MG, and found a positive association between both CRP and ESR with b2MG [25]. These findings are concomitant with our study.

SLE disease activity was calculated by the SLEADI score, which showed a positive relationship between SLEADI score and serum b2MG (*r* = 0.725, *p* < 0.001), with a significantly higher level in-group II. Kim et al., in their study on the Korean lupus population, revealed a positive association between SLEDAI and b2MG, which is concurrent to our findings [9]. Lwona et al*.* showed elevated b2MG associated with the disease activity [26]. Hermansen et al*.* also revealed a significant relationship between b2MG and SLEDAI scores [10]. According to previous studies, b2MG might be utilized as an accurate indicator for assessing SLE disease activity.

Our study revealed negative association with HGB levels (*r* = − 0.446, *p* < 0.001). This is in agreement with the findings of Skare et al., which indicated a negative correlation between serum b2MG with both HGB and C3 (*p* value 0.002, 0.007), respectively [25]. Furthermore, the study of Evrin and Ström on 23 Swedish lupus subjects revealed a negative association with HGB levels [27]. Anemia is primarily linked to LN due to impaired erythropoietin production induced by inhibiting inflammatory cytokines and serum b2MG. It is regarded as a marker for inflammation [28].

This study found a negative link between serum b2MG and C3. In contrast, there was no significant correlation with anti-dsDNA. Żychowska et al*.* also revealed a relationship between the levels of b2MG with C4 and C3 complements, but anti-dsDNA was found to be significantly correlated [29]. In contrast to our study, no substantial association was detected between serum b2MG with C3, as well as C4 in a study by Aghdashi et al. [30], which may be due to differences in ethnic and environmental factors and a small sample size of 50 patients.

Serum b2MG enhances LN progression besides inducing additional kidney damage, as detected in this study. It shows that b2MG is substantially linked to creatinine, urea levels, and proteinuria (*p* < 0.001) but is negatively linked to eGFR (*p* < 0.001). This is in accordance with the finding of Liu et al., which suggested a negative correlation between serum b2MG and GFR (*r* =  − 0.873, *p* = 0.000) [5]. Kim et al. also revealed similar results regarding the relationship between serum b2MG with BUN, serum creatinine, and proteinuria, which were significantly correlated [9].

In this study, renal, cardiac, and neurological damages were substantially higher in-group II. In addition, SLE cumulative damage measured by SLICC/ACR was positively linked to serum b2MG (*r* = 0.603, *p* < 0.001) and was more prominent in-group II (*p* < 0.001). These results concluded the role of serum b2MG not only as a disease activity index but also in determining damage score. Wang et al. demonstrated the role of serum b2MG as an effectively specific and sensitive biomarker for early monitoring of change in renal function due to secondary kidney damage [31].

In contrast to our results, Skare et al. have not revealed any link between b2MG and SLE cumulative damage assessed by SLICC/ACR [25], which can be explained by different environmental and ethnic factors. In addition, using a different method for measuring serum b2MG (utilizing chemiluminescence (Immulite 2000, Diagnostic Products Corporation, USA*)*, with normal values of 604–2786 ng/ml) may be considered as another reason for these differences.

ROC curve analysis comprehensively and accurately assessed the diagnostic performance of serum b2MG as a predictor of LN, disease activity, and cumulative damage in cases with SLE. Our study revealed that the best cutoff value of serum b2MG to predict LN is > 2.6 µg/ml, specificity of 74.47, PPV of 80.3, the sensitivity of 92.45, NPV of 89.7, with AUC of 0.874, while for prediction of disease activity, the best cutoff value was > 2.2 µg/ml with a specificity of 85, sensitivity of 96.67, PPV of 90.6, NPV of 94.4, and an AUC of 0.961. Meanwhile, for cumulative damage prediction cutoff value was > 2.6 with specificity, sensitivity, PPV, and NPV were 65.45, 93.33, 68.9, and 92.3, respectively, with an AUC of 0.836. The current findings align with Liu et al., demonstrated that kidney damage can be effectively predicted by serum b2MG levels using ROC curve analysis. According to them, AUC was 0.881, denoting an elevated predictive value. The cutoff value of b2MG was 7.48 mg/L. Meanwhile, AUC was 0.863 for predicting the disease activity of SLE, with an elevated predictive value, while the cutoff value of b2MG levels was 6.69 mg/L [5].

Huang et al. studied the role of both Anti-α-enolase antibodies along with b2MG for the prediction of LN in SLE patients and found that b2MG can be used as a predictor of LN with an AUC of 0.845. They also concluded that AUC became higher (0.927), and the prediction of LN increased when Anti-α-enolase antibody and serum b2MG are combined together [32].

Serum b2MG levels may be utilized for anticipating LN progression as well as identifying disease severity and cumulative damage for proper management with significant sensitivity and specificity.

## Limitation of the current study

Our findings need to be interpreted carefully considering the following limitations. First, our study is cross-sectional in a group of patients at a certain time and requires data from patients follow. Second, a few numbers of articles studied the relation between serum b2MG and damage score. Finally, yet importantly, the results of our study are for a certain geographical area, so these results may not be completely generalized.

## Conclusion

Serum b2MG is significantly associated with lupus nephritis, disease activity, and cumulative disease damage. In addition, it can be utilized as a useful noninvasive biomarker in SLE with elevated specificity as well as sensitivity to determine the development of LN, degree of disease activity, cumulative damage, and disease progression for proper management.

## Data Availability

The data sets used and/or analyzed during the current study are available from the corresponding author on reasonable request.
